# Supplementary material for: Hydrologic resilience and Amazon productivity
Source: Nat Commun. 2017 Aug 30;8:387. doi: 10.1038/s41467-017-00306-z (PMC5577139; doi:10.1038/s41467-017-00306-z)
Supplement: Supplementary file 1 — Supplementary Information [file 41467_2017_306_MOESM1_ESM.pdf]

Title: Supplementary Information

Description: Supplementary Table and Supplementary Figures

Title: Peer Review File

Description:

Supplementary Table 1. CMIP5 models and modelling groups. ESMs used in full analysis are marked with bold font.

**Modeling Center (or Group)**	**Institute ID**	**Model Name**
Canadian Centre for Climate Modelling and Analysis	CCCMA	**CanESM2**
National Center for Atmospheric Research	NCAR	**CCSM4**
Community Earth System Model Contributors	NSF-DOE-NCAR	**CESM1(BGC)**
Centre National de Recherches Météorologiques / Centre Européen de Recherche et Formation Avancée en Calcul Scientifique	CNRM-CERFACS	CNRM-CM5
Commonwealth Scientific and Industrial Research Organization in collaboration with Queensland Climate Change Centre of Excellence	CSIRO-QCCCE	CSIRO-Mk3.6.0
EC-EARTH consortium	EC-EARTH	EC-EARTH
LASG, Institute of Atmospheric Physics, Chinese Academy of Sciences	LASG-IAP	FGOALS-s2
NOAA Geophysical Fluid Dynamics Laboratory	NOAA GFDL	GFDL-CM3<br>**GFDL-ESM2M**
NASA Goddard Institute for Space Studies	NASA GISS	GISS-E2-R
Met Office Hadley Centre	MOHC	HadGEM2-CC<br>**HadGEM2-ES**
Institute for Numerical Mathematics	INM	INM-CM4
Institut Pierre-Simon Laplace	IPSL	IPSL-CM5A-LR<br>**IPSL-CM5A-MR**
Japan Agency for Marine-Earth Science and Technology, Atmosphere and Ocean Research Institute (The University of Tokyo), and National Institute for Environmental Studies	MIROC	**MIROC-ESM**<br>**MIROC-ESM-CHEM**
Atmosphere and Ocean Research Institute (The University of Tokyo), National Institute for Environmental Studies, and Japan Agency for Marine-Earth Science and Technology	MIROC	MIROC5
Max-Planck-Institut für Meteorologie (Max Planck Institute for Meteorology)	MPI-M	**MPI-ESM-LR**
Meteorological Research Institute	MRI	MRI-CGCM3
Norwegian Climate Centre	NCC	NorESM1-M

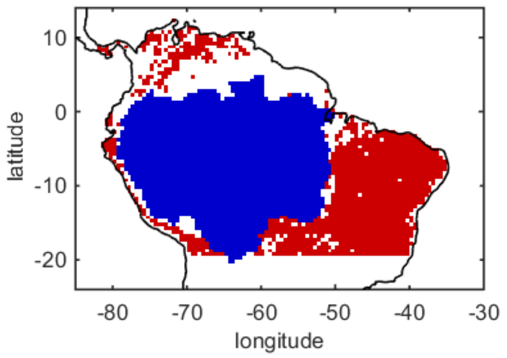

Supplementary Figure 1. Geographical boundary of the Amazon basin (green) and surrounding semi-arid regions (red).

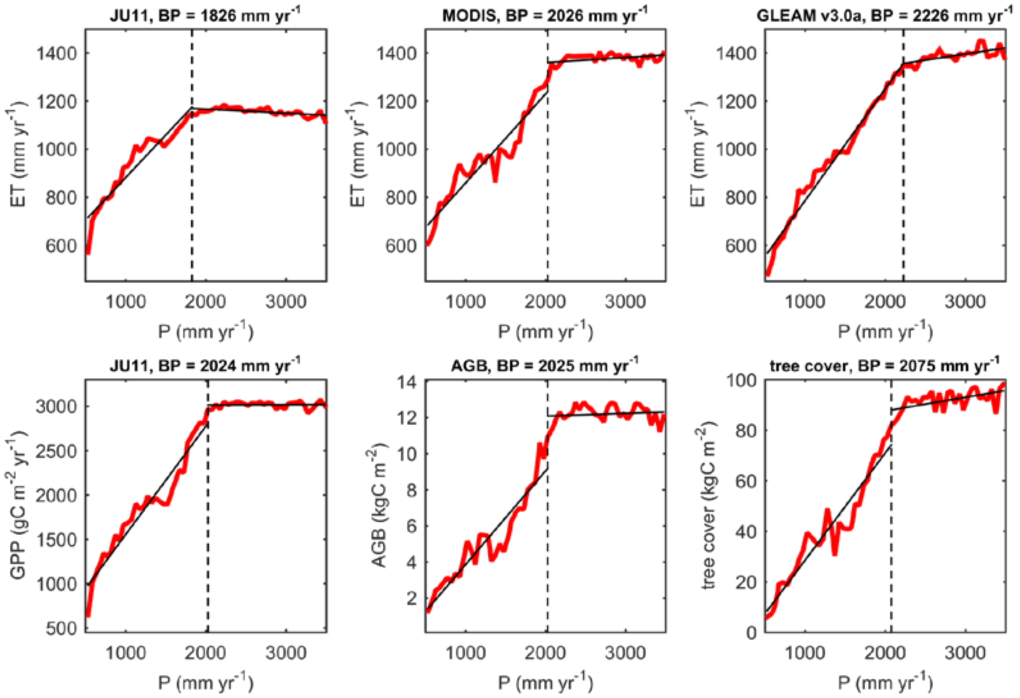

Supplementary Figure 2. Trend break analysis of six empirical datasets. The figure shows the results of estimating the breakpoint through a trend break analysis using two linear curves. Red lines represent the functional relationship found by averaging ET, GPP, AGB and tree cover over 50mm precipitation bins. We estimated two linear curves with intercepts by minimization of the sum of squared errors within the 500-3500mm P range. This 50mm bin size is the reason for why the estimated breakpoints has a values close to xx25 and xx75 mm yr^{-1}, which represent the mean P within each bin. The influence of land use change (see main paper) influences the intersection of the two curves and explains parts of the offsets between them at the breakpoint (e.g. for AGB).

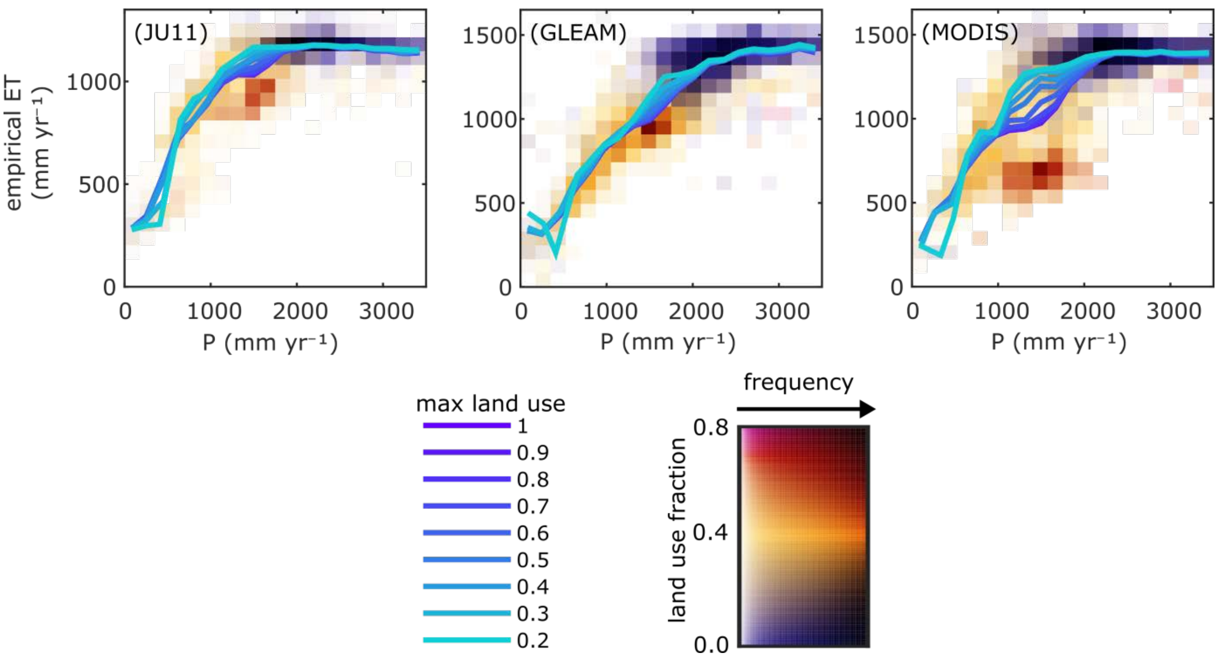

Supplementary Figure 3. Functional P-ET relationships and their dependency on land use. The figure complements Figure 4 in main paper. Coloured frequency field shows the dependency of ET on P and land use. Colour follows the mean land use fraction in a P-ET bin, and the saturation describes relative frequency. Coloured lines represent the functional relationships, found when averaging ET over P-bins. The colours describe the maximum land use fraction of the gridcells included in the calculations of the functional relationships; from all gridcells (1) to only include gridcells with land use fraction lower than 0.2.

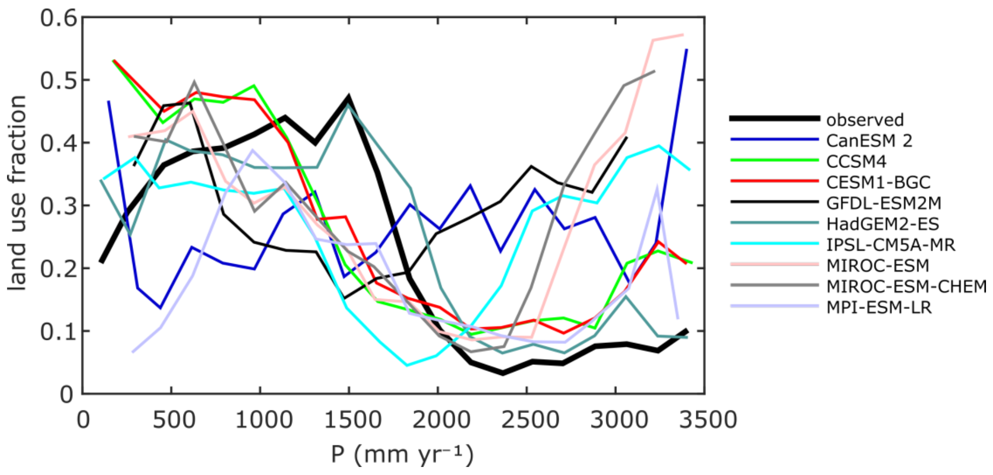

Supplementary Figure 4. Relationship between P and land use. Only HadGEM2-ES accurately captures the peak of land use at 1500 mm annual P.

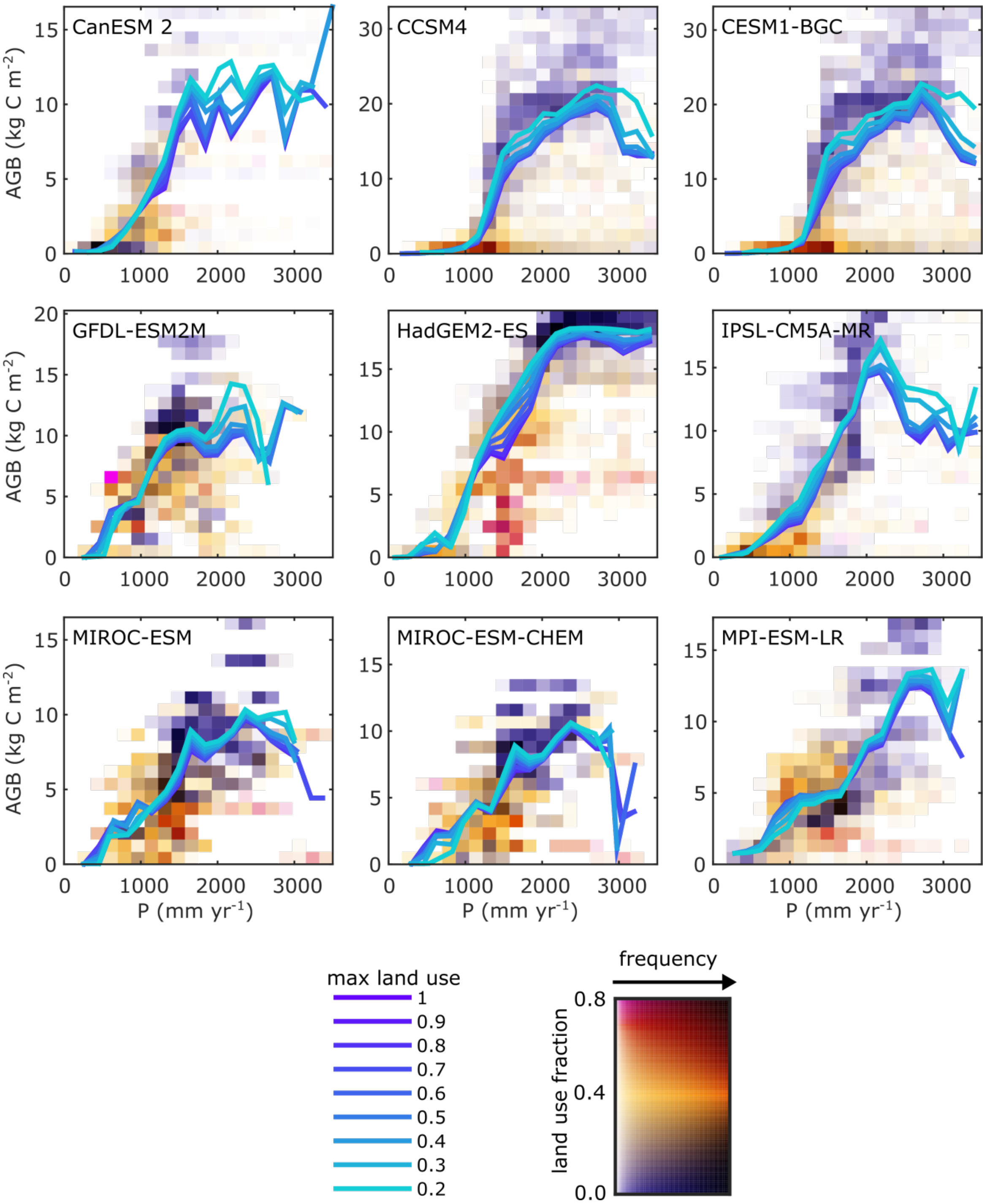

Supplementary Figure 5. ESM functional relationships and their dependency on land use. Panels show the dependency of AGB on P in nine ESMs. Coloured frequency field shows the dependency of AGB on P and land use. Colour follows the mean land use fraction in a P-AGB bin, and the saturation describes relative frequency. Coloured lines represent the functional relationships, found when averaging AGB over P-bins. The colours describe the maximum land use fraction of the gridcells included in the calculations of the functional relationships; from all gridcells (1) to only include gridcells with land use fraction lower than 0.2.

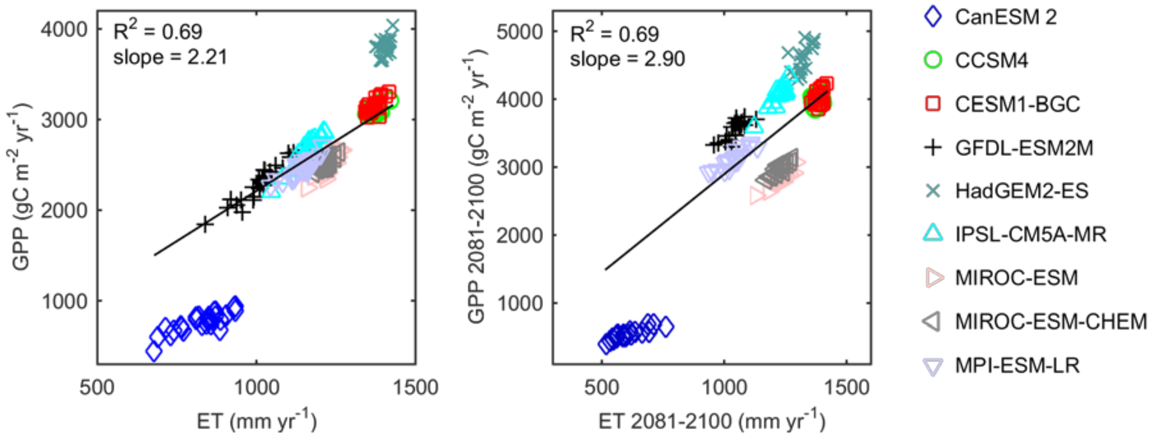

Supplementary Figure 6. Amazon basin eWUE (slope) for the (a) historical (1981-2005) and (b) future (2081-2100) period. The intercept of the regression is forced to zero.

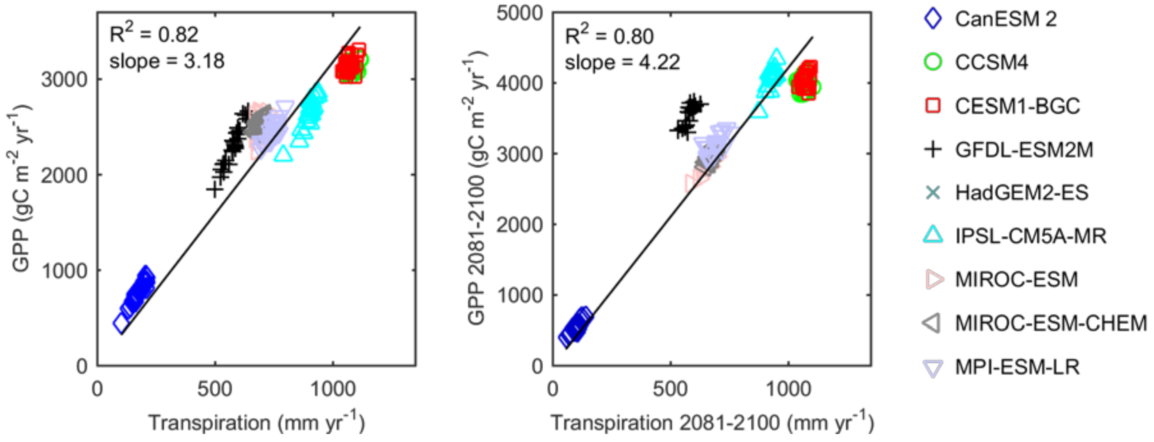

Supplementary Figure 7. Amazon basin WUE for the (a) historical (1981-2005) and (b) future (2081-2100) period. The intercept of the regression is forced to zero.

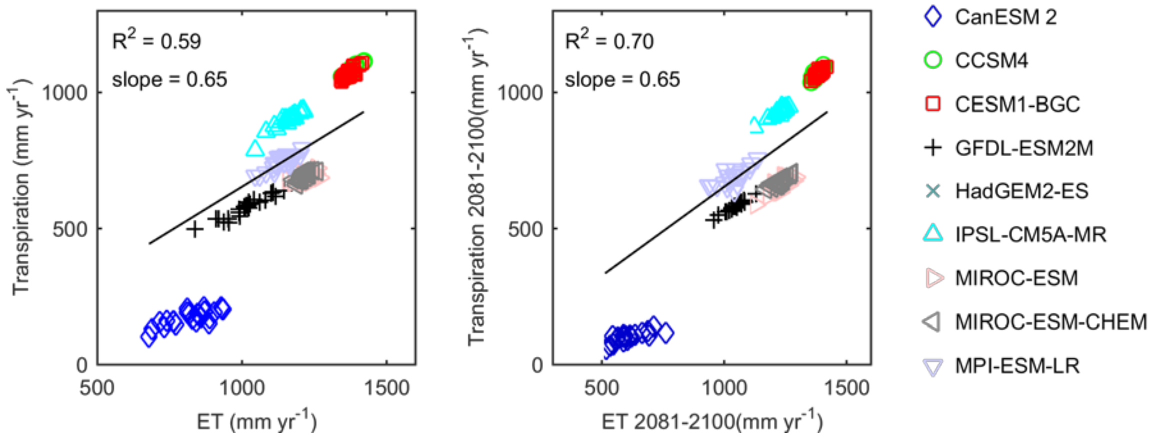

Supplementary Figure 8. Amazon basin transpiration and ET for the (a) historical (1981-2005) and (b) future (2081-2100) period. The intercept of the regression is forced to zero.

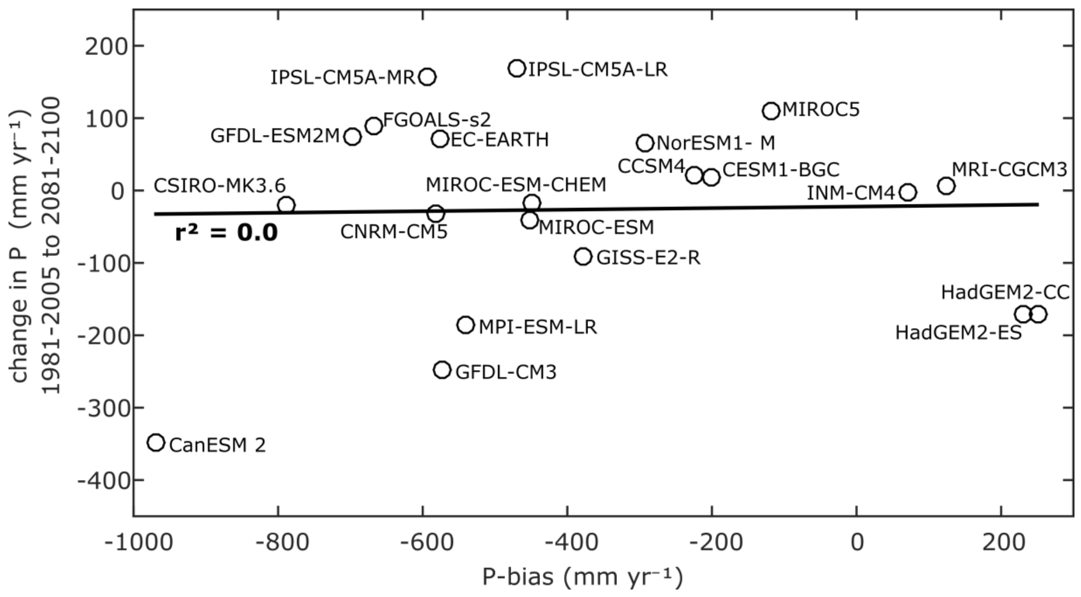

Supplementary Figure 9. Concurrent climate bias and future change in Amazon basin P. The figure shows that there is no emergent relationship between the ESMs climate bias and their predicted future changes, limiting the ability to constrain future Amazon P.
